# Rapid detection of 2-hydroxyglutarate in frozen sections of IDH mutant tumors by MALDI-TOF mass spectrometry

**DOI:** 10.1186/s40478-018-0523-3

**Published:** 2018-03-02

**Authors:** Rémi Longuespée, Annika K. Wefers, Elena De Vita, Aubry K. Miller, David E. Reuss, Wolfgang Wick, Christel Herold-Mende, Mark Kriegsmann, Peter Schirmacher, Andreas von Deimling, Stefan Pusch

**Affiliations:** 1 Institute of Pathology, Ruprecht-Karls-University Heidelberg, Im Neuenheimer Feld, 224 Heidelberg, Germany; 2 Department of Neuropathology, Institute of Pathology, Ruprecht-Karls-University Heidelberg, Im Neuenheimer Feld, 224 Heidelberg, Germany; 3 German Consortium of Translational Cancer Research (DKTK), Clinical Cooperation Unit Neuropathology, German Cancer Research Center (DKFZ), Im Neuenheimer Feld, 280 Heidelberg, Germany; 4 Cancer Drug Development, German Cancer Research Center (DKFZ), Im Neuenheimer Feld, 280 Heidelberg, Germany; 5 Clinical Cooperation Unit Neurooncology, German Cancer Consortium (DKTK), German Cancer Research Center (DKFZ), Im Neuenheimer Feld, 280 Heidelberg, Germany; 6 Department of Neurology, Heidelberg University Hospital, Im Neuenheimer Feld, 400 Heidelberg, Germany; 7 Division of Experimental Neurosurgery, Department of Neurosurgery, Heidelberg University Hospital, Im Neuenheimer Feld, 400 Heidelberg, Germany

**Keywords:** *IDH* mutation, 2-hydroxyglutarate, Diffuse glioma, MALDI-TOF, Mass spectrometry

## Abstract

All isocitrate dehydrogenase (IDH) mutant solid neoplasms exhibit highly elevated levels of D-2-hydroxyglutarate (D-2HG). Detection of 2HG in tumor tissues currently is performed by gas or liquid chromatography-mass spectrometry (GC- or LC-MS) or biochemical detection. While these methods are highly accurate, a considerable amount of time for tissue preparation and a relatively high amount of tissue is required for testing. We here present a rapid approach to detect 2HG in brain tumor tissue based on matrix-assisted laser desorption ionization - time of flight mass spectrometry (MALDI-TOF). We analyzed 26 brain tumor samples with known *IDH1* or *IDH2* mutation and compared readouts to those from 28 brain tumor samples of wildtype IDH status. IDH mutant samples exhibited a clear positive signal for 2HG which was not observed in any of the IDH wildtype tumors. Our analytical pipeline allowed for 2HG detection in less than 5 min. Data were validated by determining 2HG levels in all tissues with a biochemical assay. In conclusion, we developed a protocol for rapid detection of 2HG levels and illustrate the possibility to use MALDI-TOF for the detection of metabolites on frozen tissue sections in a diagnostic setting.

**Electronic supplementary material:**

The online version of this article (10.1186/s40478-018-0523-3) contains supplementary material, which is available to authorized users.

## Introduction

Mutations of the isocitrate dehydrogenase genes (*IDH1* and *IDH2*) have been detected in several tumor types including chondrosarcoma, acute myeloid leukemia (AML), cholangiocarcinoma and diffuse glioma [1, 2, 5, 18, 25]. Especially in the diagnosis of brain tumors, the IDH mutation status has gained a dominant role for classification [16]. IDH mutations are present in astrocytoma and oligodendroglioma but absent in other glial/glioneuronal tumors entities constituting differential diagnoses [21].

Mutations predominantly occur in codons 100 and 132 of *IDH1* and codons 140 and 172 of *IDH2*. All these mutations result in a change of substrate specificity. While wildtype IDH decarboxylates isocitrate to α-ketoglutarate (αKG), mutant IDH1 and IDH2 reduce αKG to D-2-hydroxyglutarate (D-2HG). This results in a dramatic increase of D-2HG up to 1000-fold in tumor cells carrying an IDH mutation. Therefore, increased levels of D-2HG in tumors can serve as a surrogate marker for mutations either in *IDH1* or in *IDH2* [9]. D-2HG is considered to be an “oncometabolite”, because it is capable to inhibit αKG-dependent enzymes which are important for diverse cellular processes, mainly epigenetic control.

Detection of 2HG in tissues so far has been performed either by LC-MS or by a biochemical assay [3, 9]. While LC-MS has the potential to detect many metabolites in a single sample, the biochemical detection method is restricted to detecting D-2HG only. However, it allows parallel analyses of a large number of samples. The sensitivity of both methods is comparable, but both require time consuming sample preparation. Detection of 2HG in vivo has been facilitated by magnetic resonance spectroscopy (MRS). However, a widely distributed detection of 2HG by spectroscopy appears not to be imminent due to limited compatibility of MRI-sequences with the hardware available and due to complex post-processing.

Mass spectrometry was recently proposed as another approach to analyze brain tumors intraoperatively. Desorption electrospray ionization (DESI)-MS was used for the rapid determination of brain tumor margins in the course of surgery, based on lipid signatures [10, 19]. 2HG was also used as a surrogate maker of the presence of IDH-mutant cancer cells for tumor margins resection using DESI-MS [19, 23].

The relatively low ionization yields for 2HG using conventional matrices for matrix assisted laser desorption ionization (MALDI) time of flight (TOF)-MS limited its use for diagnosis compared to DESI-MS.

Herein we present an approach to detect 2HG in frozen sections of brain tumor tissues by MALDI-TOF MS within less than 5 min. The proposed approach is a proof of concept study to show how MALDI-TOF MS could support diagnostic decisions on minute preparations.

## Material and methods

### Material

All solvents were purchased from Thermo Fisher Scientific (Waltham, USA). The indium thin oxide (ITO)-coated glass slides were obtained from Bruker Daltonik (Bremen, Germany). The MALDI matrices as well as pure metabolite compounds were purchased from Sigma-Aldrich (Taufkirchen, Germany). The 10 μl tips and microloader tips were purchased from Eppendorf (Hamburg, Germany).

### Tissue samples

Fresh frozen tumor tissues from 54 patients with predetermined IDH status were selected from the archive of the Department of Neuropathology, Heidelberg. Of those, 26 tumor tissues carried either an *IDH1* or an *IDH2* mutation, whereas the other 28 tumor tissues were *IDH1/2* wildtype and served as negative test tissue (Additional file 1: Table S1). The series included 11 diffuse astrocytomas WHO grade II (DA), 4 anaplastic astrocytomas WHO grade III (AA), 7 oligodendroglioma (O), 3 anaplastic oligodendrogliomas (AO), 1 pilocytic astrocytomas WHO grade I (PA), 1 ganglioglioma WHO grade I (GG), 12 glioblastoma WHO grade IV (GBM), 13 schwannoma WHO grade I, and 1 non-small cell lung cancers (NSCLC). Of the IDH mutant DA, AA, O, AO and GBM 19 contained an IDH1R132H, 1 an IDH1R132C, 1 an IDH1R132G, 1 an IDH1R132S, 2 an IDH2R172K, 1 an IDH2R172S and 1 an IDH2R172M mutation.

**Fig. 1:**
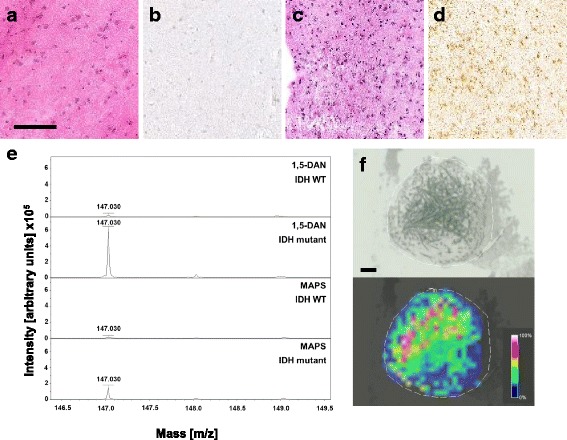
Pre-characterization and 2HG quantification on tissue samples. **a** H&E staining of IDH wildtype test tissue (CNS-tissue with reactive change), the size bar represents 500 μm. **b** IDH1R132H staining of IDH wildtype tissue shows no IDH1R132H positive cell. **c** H&E staining of IDH mutant test tissue (diffuse astrocytoma, WHO grade II). **d** IDH1R132H staining of test tissue shows IDH1R132H positive cells. **e** MALDI-TOF spectra showing m/z values from 146.5 to 149.5 of IDH wildtype and IDH mutant tissues with the matrices 1,5-DAN and MAPS. Peaks at m/z 147 correspond to 2HG. **f** Light microscopic image and corresponding MALDI-TOF image of a representative 0.05 μl 1,5-DAN spot on IDH mutant tissue. The size bar represents 150 μm, the intensity bar shows the color coding for MALDI-TOF intensities within the scanned region

Cases for analysis of the IDH-status via detection of 2HG were selected according to the following criteria: 1) knowledge of IDH-status, 2) tissue size sufficient for repeated analyses, 3) sufficient viable tumor tissue contained. For IDH wildtype samples, most tissues were selected from different brain tumor subtypes and one CNS tissue with reactive change. For IDH-mutant cases, gliomas with different IDH-mutations were collected. Examples for pre-characterization of tissues are shown in Fig. 1a-d. All samples were analyzed in an anonymous way. Informed consent has been provided in accordance with the local ethics committee.

### Morphological analysis and immunohistochemistry

Prior to inclusion of samples, immunohistochemical staining for IDH1 R132H-mutant protein have been performed as described previously [7].

From archived tissue, frozen sections were cut to 4 μm with a Leica CM 3050 S cryostat (Leica Biosystems, Nussloch, Germany) in a defined orientation. H&E stainings from all frozen sections were analyzed microscopically to ensure the presence of vital tumor cells. Vital areas were marked on the slides for orientation in the MALDI-TOF-assay.

### Detection of rare IDH mutations by sequencing

Prior to inclusion of samples, *IDH1* exon 4 encompassing codon 132 and *IDH2* exon 4 encompassing codon 172 have been subject to analysis by direct sequencing using an ABI 3100 DNA analyzer (Thermo Fisher Scientific, Waltham, USA) as previously described [13].

### D-2HG detection by biochemical assay

The D-2HG assay has been described previously [3]. In brief, three 10 μm-thick slices were dissolved in 125 μl cell lysis buffer (150 mM NaCl, 0.1% NP-40, 50 mM Tris-HCl, pH 8.0) and subsequently treated with a deproteinization kit (Biovision, Mountain View, CA, USA). Supernatants were then collected and stored at − 20 °C. The total enzymatic reaction volume was 100 μl. Ten milliliters of assay solution were freshly prepared for each 96-well plate subjected to D-2HG assay. The assay solution contained 100 mM HEPES pH 8.0, 100 μM NAD^+^, 5 μM resazurin (Applichem, Darmstadt, Germany), 0.1 μg HGDH and 0.01 U/ml diaphorase (0.01 U/ml; MP Biomedical, Irvine, USA). Immediately before use, 25 μl sample volume was added to 75 μl of assay solution and incubated at room temperature for 30 min in black 96-well plates (Thermo Fisher Scientific, Waltham, USA) in the dark. Fluorometric detection was performed in triplicate with 25 μl deproteinized sample being analyzed in each reaction with excitation at 540 ± 10 nm and emission of 610 ± 10 nm (FLUOstar Omega, BMG Labtech, Offenburg, Germany).

### Maleic anhydride proton sponge (MAPS) synthesis

MAPS was synthesized according to previously reported procedures [12, 24]: A solution of 1,8-Bis(dimethylamino)naphthalene (1.1 ml, 12 mmol – Sigma-Aldrich) in anhydrous THF (35 ml) was added to an orange solution of bromovaleric anhydride (5.0 g, 24 mmol – Sigma-Aldrich) in anhydrous THF (20 ml) under Argon at room temperature, immediately producing a deep red suspension. After 1 h, the suspension was concentrated in vacuo, redissolved in THF (60 ml) and filtered through paper. The filtrate was concentrated to give a purple-blackish crystalline solid (3.5 g, 95% yield).

^1^H NMR (400 MHz, CDCl_3_) δ: 7.90 (d, *J* = 8.5 Hz, 1H), 7.56 (d, *J* = 8.5 Hz, 1H), 7.40 (t, *J* = 8.0 Hz, 1H), 6.95 (d, *J* = 7.6 Hz, 1H), 6.87 (s, 1H), 6.85 (d, *J* = 8.1 Hz, 1H), 2.95 (s, 6H), 2.80 (s, 6H).

^13^C NMR (101 MHz, CDCl_3_) δ: 166.7, 165.4, 154.9, 151.8, 146.2, 136.3, 132.0, 128.2, 127.0, 121.6, 117.8, 116.2, 115.4, 112.5, 109.5, 43.4, 43.3.

HRMS (ESI) m/z: (M + H)^+^ calcd for C_18_H_19_N_2_O_3_: 311.1390; found: 311.1391.

### Matrix solubilization and deposition on tissues

Four μm thick frozen sections were cut and thaw mounted onto ITO glass slides. Each slide contained both *IDH* wildtype and *IDH*-mutant sections. Brain tumor sections were dried at room temperature for 1 min.

Dihydroxybenzoic acid (DHB) was dissolved in a mixture of ACN/aqTFA 0.1% 7:3 at a concentration of 14 mg/ml. 9-amino acridine (9-AA) was dissolved in a mixture of MeOH/H_2_O 7:3 at a concentration of 10 mg/ml. 1,5-diaminonaphtalene (1,5-DAN) was dissolved in a mixture of ACN/aqTFA 0.1% 7:3 at a concentration of 6 mg/ml.

Different solvent mixtures were tested for the solubilization of 5 mg/ml of MAPS: ACN/aqTFA 0.1% 7:3, ACN/aqTFA 0.1% 9:1 and ACN/Chloroform 9:1.

The solutions were manually deposited on top of the regions of interest of the tissues, using a micropipette and 0.5–10 μl classical tips or microloader tips (Eppendorf, Wesseling-Berzdorf, Germany).

### 2HG profiling in tissues

Detection of 2HG in tissues was performed using the Rapiflex MALDI-TOF mass spectrometer (Bruker Daltonik, Bremen, Germany) which is equipped with a smartbeam laser (Nd:YAG 355 nm) operating at 10,000 Hz. The laser was set in MS dried droplet. MALDI analyses were operated in the reflector negative mode in order to detect the [M-H]- species of 2HG at m/z 147. The following settings were used: mass range analyzed: m/z 0–740, ions source 1 voltage: 19.87 kV, PIE: 2.417 kV, lens: 11.672, reflector 1: 20.835 kV, reflector 2: 1.01 kV, reflector 3: 8.58 kV, detector gain: 3135 V, sample rate 5GS/s, analog offset: 70.1 mV, global attenuator offset: 14%, laser intensity: 70%, movement on samples spot: off, matrix suppression: deflector. The calibration was made in negative mode using maleic acid (m/z 115.01), glutaric acid (m/z 131.04), alpha ketoglutarate (m/z 145.02), ascorbic acid (m/z 175,03) and isocitric acid (m/z 191.03).

### MALDI imaging of 1,5-DAN spots

MALDI imaging was performed using a raster step of 50 μm. 5000 shots were acquired per spot and images dataset were constructed using flex imaging (Bruker Daltonik, Bremen, Germany).

### Statistical methods

All statistical analysis was performed with Sigma Plot Version 13.0. The corresponding test used for the analysis is depicted in the figure legend.

## Results

### Evaluation of commercially available matrices for detection of a D-2HG solution via MALDI-TOF

We aimed to find a commercially available matrix that allows for the ionization and desorption of 2HG. So far, only one matrix was reported to be suitable for the MALDI analysis of 2HG in tissues using MALDI-TOF instrumentations. This matrix, MAPS, is however not commercially available [12].

We tested three commercially available matrices for their capability to detect 2HG (Additional file 2: Figure S1a). We analyzed 0.3 μl spots containing 10 mM of D-2HG. This concentration is the mean 2HG level found in IDH-mutant brain tumor tissues [20, 22]. We used matrices that were previously used for the analysis of small molecules and metabolites, namely 2,5-dihydroxydenzoic acid (DHB) [11] and 9-aminoacridine (9AA) [17]. 1,5-diaminonaphtalene (1,5-DAN) [15], a known basic matrix was also tested as it could be adequate for the deprotonization of the acidic 2HG.

We could reliably detect 2HG only in negative mode at m/z 147 using all the matrices, at different intensities (Additional file 2: Figure S1a). The intensity of 2HG using 9-AA was weaker when compared to 1,5-DAN.

No background signal was observed from the matrices as shown in the controls when matrices alone were analyzed (Additional file 2: Figure S1a). Given the high reliability of 1,5-DAN to detect 2HG, we further continued our experiments using this matrix.

### Evaluation of the matrices MAPS and 1,5-DAN for detection of 2HG in test tissues via MALDI-TOF

We evaluated the capacity of 1,5-DAN to detect 2HG in IDH mutant brain tumor tissues, and compared it with the non-commercially available matrix MAPS [12]. MAPS was developed to induce the negative ionization of 2HG in the liquid phase. We attempted to dissolve MAPS in the same solution as 1,5-DAN (ACN/TFA 0.1% 7:3), but the high hydrophobicity of the matrix did not allow the use of an aqueous solution. We then tested the mixture ACN/TFA 0.1% 9:1 but the solubilization of MAPS was not improved. Only the mixture ACN/Chloroform 9:1, as previously described, [12] was successful for the solubilization of MAPS.

We selected one IDH wildtype and one IDH mutant tissue to compare the two matrices. For this first test, droplets of 0.3 μl were deposited onto tissues, as this volume is easy to handle with standard tips.

Both matrices allowed us to detect 2HG in tissue. However, we could retrieve more than 3-fold higher intensities of 2HG from tissues using 1,5-DAN compared to MAPS (Fig. 1e). For this reason, we used 1,5-DAN for all further experiments.

### MALDI imaging of 2HG with 1,5-DAN spots in test tissues

We aimed to determine where the highest signals of 2HG could be found in 1,5-DAN spots. This knowledge would help targeting specific crystals of 1,5-DAN for analyses.

We performed MALDI imaging of 0.3 μl 1,5-DAN spots on the test tissues. The results taught us that the thinnest “hairy” crystals at the center of the spots allowed retrieving the highest signal from 2HG. The crystals appeared white and opaque by eye and through the camera of the MALDI-TOF instrument. The opacity of the white hairy crystals made these appear black on optical scans (Additional file 2: Figure S1b).

### MALDI imaging of 2HG with smaller 1,5-DAN spots in test tissues

The small size of tumor areas in some biopsies may require depositing very small 1,5 DAN spots in order to retrieve specifically the signal from cancerous regions. We checked if smaller spots could provide a sufficient extraction of 2HG from the tissues while ensuring the particular crystallization where highest intensities of 2HG can be detected. The minimum volume we could set using micropipettes was 0.05 μl. Pipetting this volume using standard 10 μl tips was difficult and resulted in different spot sizes. To circumvent this we used microloader tips. We first tested the reproducibility of the spots and observed that their size was similar between triplicates (diameter about 1 mm against 2 mm for 0.3 μl spots).

The small spots showed the intended thin and opaque 1,5-DAN crystals (Fig. 1f). These could clearly be recognized through the camera of the MALDI-TOF instrument. MALDI imaging revealed that these crystals allowed retrieving the highest signals of 2HG.

### Analysis of 2HG with 1,5-DAN in a tissue validation set with MALDI-TOF

**Fig. 2:**
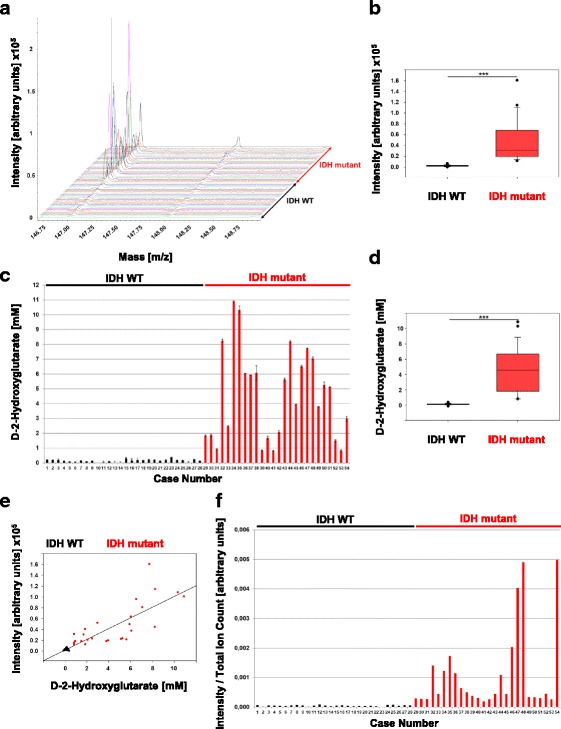
2HG quantification of validation set. **a** MALDI-TOF spectra showing m/z values from 146.75 to 148.75 of IDH wildtype and IDH mutant tissue with the matrices 1,5-DAN. Peaks at m/z 147 represent 2HG. The colors of the spectra correlate with the colours shown in Additional file 1: Table S1. **b** Box plots of MALDI-TOF signal intensities of IDH wildtype and IDH mutant tissue at m/z 147. Statistical analysis was performed with Wilcoxon-Mann-Whitney-Test (*p* < 0.0001). Error bars represent the standard deviation. **c** D-2HG concentrations of the validation set measured by the biochemical assay. **d** Box plots of D-2HG concentrations of IDH wildtype and IDH mutant tissue. Statistical analysis was performed with Wilcoxon-Mann-Whitney-Test (*p* < 0.0001). Error bars represent the standard deviation. **e** Correlation plot of MALDI-TOF intensities and biochemical determined D-2HG concentrations. Correlation was determined by Pearson (*r* = 0.862, *p* < 0.0001). **f** Normalized MALDI-TOF values of validation set. Values shown are intensities divided by the total ion count of each corresponding spectra

To validate the MALDI-TOF method for 2HG detection on tissue, we used a collection of 26 IDH mutant tumor samples and 28 tissue samples with wildtype IDH status. The overall size of tumor areas in the tissues allowed us to drop slightly larger matrix spots. We applied single droplets of 0.1 μl of 1,5-DAN onto the area of interest. Within each drop, we measured 4 different spots of 5000 laser shots, resulting in spectra from 20,000 laser shots. We detected a distinctive higher intensity of 2HG in IDH mutant tissues compared to IDH wildtype samples (Fig. 2a). 2HG was strongly increased in mutant tumors irrespectively of the type of the *IDH1* or *IDH2* mutations.

### Quantification of D-2HG by biochemical assay and comparison to MALDI-TOF data

To validate our MALDI-TOF analysis and compare it to an established assay, we quantified D-2HG levels in the tissues with our biochemical assay (Fig. 2c).

Next, we analyzed the data of both measurements in more depth, by comparing the two groups IDH wildtype with IDH mutant. For MALDI-TOF we analyzed the signal intensity at m/z 147 (Fig. 2b) and for the biochemical assay we used the D-2HG concentration (Fig. 2d). Both methods can significantly distinguish IDH wildtype from IDH mutant tissue (Fig. 2b and d).

Although absolute quantification by MALDI-TOF is not possible without internal labeled standard, we aimed to find a correlation between MALDI data and the biochemical assay (Fig. 2e). A strong positive correlation could be found between MALDI intensities of m/z 147 of each spectrum and the quantities of 2HG defined by the biochemical assay (r = 0,862). We further tried to normalize the intensity data to allow a reliable statement about IDH status, without the use of an internal standard. Therefore, we divided the intensity at 147 m/z by the total ion count (TIC) (Fig. 2f). The TIC corresponds to the overall signal of the compounds detected in the tissues. The number and the intensities of the compounds in tissues can greatly impact on the detection of a given compound of interest. This effect is called ion suppression. Since the overall signals were different between spectra of the different samples, it may have impacted differently on the final detection of 2HG. Also, MS intensities can slightly vary from an instrument to another. Dividing the intensities of 2HG by the TIC would give a more representative value corrected for ion suppression effects and instrumental variations.

Analysis of these results showed that IDH wildtype samples now could reliably be distinguished from IDH mutant samples by at least factor 2.

### Real time test on a frozen section

**Fig. 3:**
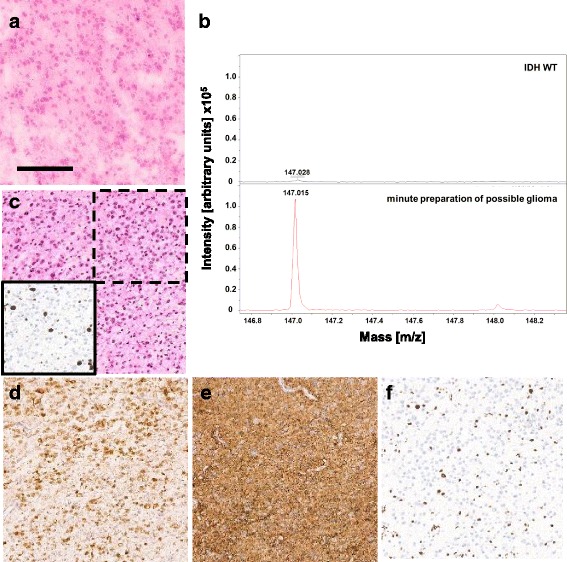
2HG measurement in real time. **a** H&E of frozen section, scale bar represents 150 μm. **b** MALDI-TOF spectra showing m/z values from 146.8 to 148.2 of one IDH wildtype as negative control and the minute preparation tissue with the matrix 1,5-DAN. Peaks at m/z 147 correspond to 2HG. **c** H&E staining of FFPE-tissue of the same case. Lower left box shows the Ki67 staining of the upper right dashed box. **d** IDH1R132H staining of the tissue shows IDH1R132H positive tumor cells. **e** GFAP staining of the tissue shows positive tumor cells. **f** ATRX staining shows loss of ATRX staining in tumor cells

To test whether our MALDI-TOF assay is feasible in a diagnostic setting, we tested the assay on a frozen section of a glioma (Fig. 3a). The time required after receiving the frozen section to obtaining the 2HG data, was 4 min and 39 s. In this case we were able to detect high amounts of 2HG (Fig. 3b, signal intensity 111,382). The high 2HG concentrations could also be validated with the biochemical assay, where this case showed a D-2HG concentration of 3.12 mM. Subsequent immunohistochemical and molecular analysis confirmed an IDH1R132H mutation in this tumor (Fig. 3c-f).

## Discussion

Rapid analysis of molecular parameters is constantly gaining importance for diagnostics and therapy of tumors. Employing the considerable increase of 2HG in IDH mutant brain tumors we demonstrate the feasibility of rapid detection of metabolites in fresh frozen sections by MALDI-TOF analysis.

The current WHO guidelines demand establishing the IDH mutation status for precise classification of diffuse gliomas [16]. This is routinely accomplished by immunohistochemical analysis with antibody H09 detecting the IDH1R132H mutation [7]. However, in diffuse gliomas approximately 10% of IDH mutations evade detection by immunohistochemistry. These tumors harbor either rarer variants of IDH1R132 mutations or mutations in *IDH2*. Thus, additional analyses are required to identify potential mutations in diffuse glioma not binding antibody H09. Typically, this is performed by Sanger- or by cycle sequencing of the respective regions of the *IDH1* and *IDH2* genes. An alternative approach has been opened since the discovery that all tumor relevant mutations in *IDH1* and *IDH2* result in a neomorphic enzyme activity ultimately leading to a dramatic increase of intracellular 2HG [9]. 2HG can be detected by LC-MS and if elevated serve as surrogate marker for IDH mutations. Subsequently a biochemical assay has been developed detecting 2HG with a comparable sensitivity [3]. Our present approach follows the same concept of detecting elevated 2HG by MALDI-TOF analysis as a surrogate for IDH mutations.

We analyzed 26 diffuse gliomas with established positive IDH status and compared to 28 tumors tested negative for IDH mutations by MALDI-TOF analysis. All IDH mutated gliomas exhibited clearly raised signals for 2HG while all control tissues exhibited only basal levels (Fig. 2a). A clear cutoff level of 12,100 could be established in our setting with all IDH mutant tumors positioned above and all IDH wildtype tissues positioned below. The data from MALDI-TOF analysis were validated by a biochemical assay [3]. We detected full agreement of both methods in all samples of our series (Fig. 2e). Further, the intensities of 2HG were normalized by the TIC. Dividing the 2HG intensity by TIC resulted in a value at least 2 times higher in tumors with IDH mutation than in IDH wildtype tissues (Additional file 1: Table S1). Alternatively, addition of labeled D-2HG to the matrix as internal standard could be used for quantification of 2HG. Nevertheless, the current setup allowed us to significantly distinguish IDH wildtype from IDH mutant tumors.

In contrast to previous assays, MALDI-TOF can be performed rapidly in an intraoperative diagnostic setting. In fact, while our series was retrospective employing stored fresh frozen tumor tissues, one case was analyzed during routine intraoperative tumor diagnostics. So while one frozen section was prepared for a H&E staining, an additional section was overlaid with the matrix 1,5-DAN. Subsequent analysis by MALDI-TOF demonstrated high 2HG levels compatible with the intraoperative diagnosis of a diffuse glioma. The preparation and MALDI-TOF analysis were completed within five minutes. In addition, MALDI-TOF analysis requires only very small amounts of tissue which is prepared similarly to that used in standard minute preparations. Spotting of multiple matrices with nano/p devices on the same section would allow testing of different metabolites or proteins. Employing MALDI-TOF for the detection of 2HG during intraoperative diagnostics may currently not be the highest priority. However, it clearly helps to validate and specify a tumor diagnosis. More importantly our example demonstrates that MALDI-TOF can be employed in intraoperative diagnostics and given to its high versatility can be used for the detection of other metabolites or characteristic marker proteins. Further, the investment for hardware is quite beyond current possibilities in many diagnostic centers. However, the operating costs for an individual test are very low making MALDI-TOF analysis an attractive tool in a future setting with multiple tests being demanded during intraoperative diagnostics.

A central requirement for specific MALDI-TOF testing is the availability of a suitable MALDI matrix. Since 2HG is an acidic and hydrophilic compound, we aimed to find a basic matrix that could be dissolved in polar solutions. We tested three well established matrices. The comparison of these matrices proved that 1,5-DAN was the most efficient for 2HG detection from pure solutions (Additional file 2: Figure S1a). The deposition of 1,5-DAN on brain tissue has already been tested and allowed the reproducible formation of crystals in spots [4]. 1,5-DAN allows C-terminal fragmentation of proteins through ion source decay (ISD) [6]. It has also been proved to be efficient for the analysis of lipids [8, 14] and metabolites [15]. The matrix MAPS has been previously used for detection of 2HG [12]. While we also detected 2HG readouts by employing MAPS, the sensitivity of 1,5-DAN was proved superior (Fig. 1e). In fact, our test proved that the detection of 2HG in tissues was about 4-fold more efficient using 1,5-DAN compared to MAPS. This was probably due to the fact that MAPS could be dissolved less efficiently in aqueous solutions, leading to a poor extraction of 2HG. Moreover, MAPS did not form crystals. This may probably lead to a lower desorption effect. Based on our MALDI-TOF imaging data, we hypothesized that the detection of 2HG in tissues using 1,5-DAN could depend on the size and the shape of the matrix crystals. We observed that the highest MALDI signal for 2HG was retrieved in thin opaque crystals in the middle of the matrix spot (Fig. 1f) that could be obtained in spots as small as 0.05 μl. We analyzed 28 IDH wildtype and 26 IDH mutant tumors. In all the tissues where the thin opaque crystals could be observed, we could reliably detect 2HG and correctly classify them according to their IDH mutation status.

While this approach based on sophisticated technology allows the determination of relevant diagnostic parameters, we want to stress that this cannot replace a skilled neuropathologist in the diagnostic process.

In conclusion, we developed a simple and quick assay for the MALDI MS-based detection of 2HG in IDH mutant brain tumor tissues. This can serve as a surrogate marker for all tumor relevant *IDH1* and *IDH2* mutations. Our approach should alert neuropathologists to the potential of MALDI-TOF analysis for efficient testing of multiple markers with diagnostic relevance.

## Additional files

Additional file 1: **Table S1.** Case information. Case number, shows the number and color code under which the corresponding tissue can be found in Fig. 2 a (color code only), c and f (number only). IDH status, shows if the tissue is considered IDH wildtype or mutant. Mutant type, gives information about the *IDH1/2* mutation status. If sequence, it is either wildtype (*IDH1/2* wildtype) or the corresponding mutation in IDH1 or IDH2 is depicted. m/z is the value of measured 2HG signal in the MALDI-TOF measurment. Signal /noise, intensity, total ion count (TIC) and intensity devided by total ion count (I/TIC) are MALDI-TOF measurement related values used for analysis. Concentration list the measured D-2HG concentration of the tissues in mM as mean from three technical replicates with corresponding standard deviation (stdev). Diameter depicts the measured diameter of the tissue sample in mm. Diagnosis, is the diagnosis following the WHO 2017 classification guidelines (CNS = central nervous system, GBM = glioblastoma, O = oligodendroglioma, AO = anaplastic oligodendroglioma, DA = diffuse astrocytoma, AA = anaplastic astrocytoma). WHO grade, WHO grading if applicable. (PDF 74 kb)

Additional file 2: **Figure S1.** Establishing of MALDI-TOF method. (a) MALDI-TOF spectra showing m/z values from 146.5 to 149.5 of solvent alone (black) and 10 mM D-2HG (red) with the matrices 9AA, 1,5-DAN and DHB. (b) Light microscopic image and corresponding MALDI-TOF scanned image of a 1 μl 1,5-DAN spot on IDH wildtype (lower) and IDH mutant (upper) tissue. The size bar represents 1 mm, the intensity bar shows the color coding for MALDI-TOF intensities within the scanned region. (PDF 196 kb)
