# Supplementary material for: Rapid detection of 2-hydroxyglutarate in frozen sections of IDH mutant tumors by MALDI-TOF mass spectrometry
Source: Acta Neuropathol Commun. 2018 Mar 2;6:21. doi: 10.1186/s40478-018-0523-3 (PMC5834865; doi:10.1186/s40478-018-0523-3)
Supplement: Supplementary file 1 — Table S1. Case information. Case number, shows the number and color code under which the corresponding tissue can be found in Fig. 2 a (color code only), c and f (number only). IDH status, shows if the tissue is considered IDH wildtype or mutant. Mutant type, gives information about the IDH1/2 mutation status. If sequence, it is either wildtype (IDH1/2 wildtype) or the corresponding mutation in IDH1 or IDH2 is depicted. m/z is the value of measured 2HG signal in the MALDI-TOF measurment. Signal /noise, intensity, total ion count (TIC) and intensity devided by total ion count (I/TIC) are MALDI-TOF measurement related values used for analysis. Concentration list the measured D-2HG concentration of the tissues in mM as mean from three technical replicates with corresponding standard deviation (stdev). Diameter depicts the measured diameter of the tissue sample in mm. Diagnosis, is the diagnosis following the WHO 2017 classification guidelines (CNS = central nervous system, GBM = glioblastoma, O = oligodendroglioma, AO = anaplastic oligodendroglioma, DA = diffuse astrocytoma, AA = anaplastic astrocytoma). WHO grade, WHO grading if applicable. (PDF 74 kb) [file 40478_2018_523_MOESM1_ESM.pdf]

Table S1

case number	IDH status	mutation type	m/z	signal/noise	intensity	TIC	I/TIC	concentration [mM]		diameter	diagnosis	WHO grade	comments
								mean	stdev	[mm]			
1	wildtype	n.d.	147,028	4	2016	28546798	7,06E-05	0,205	0,01	0,8	Schwannoma	I	
2	wildtype	n.d.	147,032	2	1186	68980951	1,72E-05	0,199	0,01	0,5	Schwannoma	I	
3	wildtype	IDH1/2 WT	147,044	5	2545	38324126	6,64E-05	0,199	0,06	5,0	PA	I	
4	wildtype	n.d.	147,040	6	3091	60155586	5,14E-05	0,127	0,01	0,7	Schwannoma	I	
5	wildtype	n.d.	147,040	5	2860	65314306	4,38E-05	0,071	0,03	0,6	Schwannoma	I	
6	wildtype	n.d.	147,031	4	2386	65452931	3,65E-05	0,073	0,00	0,7	Schwannoma	I	
7	wildtype	n.d.	147,025	5	2913	47863525	6,09E-05	0,137	0,02	0,4	Schwannoma	I	
8	wildtype	n.d.	147,250	7	3828	47380043	8,08E-05	0,092	0,01	0,5	Schwannoma	I	
9	wildtype	n.d.	147,051	5	3806	60897595	6,25E-05	0,123	0,02	0,7	Schwannoma	I	
10	wildtype	IDH1/2 WT	147,027	n.a	943	63507992	1,48E-05	-0,124	0,09	4,0	CNS-tissue	n.d.	post removal of brain metastasis of NSCLC
11	wildtype	IDH1/2 WT	147,053	n.a	3822	77954962	4,90E-05	0,050	0,07	4,0	GBM, IDH-wildtype	IV	
12	wildtype	IDH1/2 WT	147,039	8	5382	58334631	9,23E-05	0,093	0,02	3,0	GBM, IDH-wildtype	IV	
13	wildtype	IDH1/2 WT	147,044	6	3028	58895052	5,14E-05	-0,014	0,13	5,0	GBM, IDH-wildtype	IV	reccurent
14	wildtype	IDH1/2 WT	147,035	2	1577	55349373	2,85E-05	0,005	0,06	5,0	GBM, IDH-wildtype	IV	
15	wildtype	IDH1/2 WT	147,040	6	3964	78153046	5,07E-05	0,302	0,07	5,0	GBM, IDH-wildtype	IV	
16	wildtype	IDH1/2 WT	147,037	6	4260	60980504	6,99E-05	0,152	0,13	5,0	GBM, IDH-wildtype	IV	
17	wildtype	IDH1/2 WT	147,037	4	2431	55031200	4,42E-05	0,189	0,13	10,0	GBM, IDH-wildtype	IV	
18	wildtype	n.d.	147,042	3	1563	52592945	2,97E-05	0,179	0,01	0,5	Schwannoma	I	
19	wildtype	n.d.	147,044	4	2034	53058169	3,83E-05	0,219	0,03	0,5	Schwannoma	I	
20	wildtype	IDH1/2 WT	147,040	4	1928	55785640	3,46E-05	0,205	0,02	0,8	GBM, IDH-wildtype	IV	reccurent
21	wildtype	n.d.	147,049	6	4806	103763061	4,63E-05	0,138	0,01	1,5	Schwannoma	I	
22	wildtype	IDH1/2 WT	147,048	2	2233	81158333	2,75E-05	0,196	0,02	0,6	GBM, IDH-wildtype	IV	
23	wildtype	IDH1/2 WT	147,039	n.a	558	91891355	6,07E-06	0,381	0,02	0,5	GBM, IDH-wildtype	IV	
24	wildtype	n.d.	147,047	5	4811	60624503	7,94E-05	0,186	0,01	8,0	mamma carcinoma	n.d.	brain metastasis
25	wildtype	IDH1/2 WT	147,051	6	6963	79431637	8,77E-05	0,166	0,01	4,0	Ganglioglioma	I	
26	wildtype	IDH1/2 WT	147,044	3	1947	49104572	3,97E-05	-0,032	0,12	3,0	GBM, IDH-wildtype	IV	
27	wildtype	n.d.	147,028	4	1791	25277191	7,09E-05	0,245	0,01	1,0	Schwannoma	I	
28	wildtype	n.d.	147,028	4	2144	32752104	6,55E-05	0,133	0,01	0,8	Schwannoma	I	
IDH WT	wildtype	IDH1/2 WT	n.a	n.a	n.a	n.a	n.a	n.a	n.a	3,0	CNS-tissue with reactive changes	n.d.	post removal of brain metastasis of NSCLC; used as IDH WT test tissue
29	mutant	IDH1 R132C	147,022	36	40512	135854699	2,98E-04	1,835	0,05	3,0	DA, IDH-mutant	II	
30	mutant	IDH1 R132S	147,022	15	12114	43109658	2,81E-04	1,861	0,08	3,0	GBM, IDH-mutant	IV	
31	mutant	IDH1 R132H	147,030	20	18201	66766186	2,73E-04	0,929	0,03	5,0	DA, IDH-mutant	II	
32	mutant	IDH1 R132H	147,024	155	114446	81108146	1,41E-03	8,248	0,09	7,0	O, IDH-mutant	II	
33	mutant	IDH1 R132H	147,025	27	23149	51931875	4,46E-04	2,483	0,05	4,0	DA, IDH-mutant	II	
34	mutant	IDH2 R172S	147,045	105	100791	82013716	1,23E-03	10,888	0,07	2,0	AA, IDH-mutant	III	
35	mutant	IDH2 R172K	147,026	176	108533	62437245	1,74E-03	10,327	0,28	4,0	O, IDH-mutant	II	
36	mutant	IDH1 R132H	147,019	96	63516	55198583	1,15E-03	6,030	0,01	3,5	AO, IDH-mutant	III	
37	mutant	IDH1 R132G	147,033	73	49540	77233864	6,41E-04	5,916	0,01	3,5	DA, IDH-mutant	II	
38	mutant	IDH1 R132H	147,052	59	37592	74163194	5,07E-04	6,085	0,48	2,5	DA, IDH-mutant	II	
39	mutant	IDH1 R132H	147,035	37	31468	82834763	3,80E-04	0,836	0,04	4,0	DA, IDH-mutant	II	
40	mutant	IDH1 R132H	147,023	40	30460	97772018	3,12E-04	1,700	0,10	3,5	DA, IDH-mutant	II	
41	mutant	IDH1 R132H	147,043	20	15069	77395588	1,95E-04	0,822	0,03	3,0	AA, IDH-mutant	III	
42	mutant	IDH1 R132H	147,025	25	20651	77395588	2,67E-04	2,076	0,11	4,0	O, IDH-mutant	II	
43	mutant	IDH1 R132H	147,042	42	21363	47273930	4,52E-04	5,625	0,10	4,0	AA, IDH-mutant	III	
44	mutant	IDH1 R132H	147,012	78	44614	40859217	1,09E-03	8,211	0,07	6,0	DA, IDH-mutant	II	
45	mutant	IDH1 R132H	147,017	34	19733	44756867	4,41E-04	3,956	0,00	3,0	AA, IDH-mutant	III	
46	mutant	IDH1 R132H	147,011	136	95951	47218496	2,03E-03	6,513	0,06	6,0	DA, IDH-mutant	II	
47	mutant	IDH2 R172K	147,017	244	160500	39765170	4,04E-03	7,718	0,03	3,5	DA, IDH-mutant	II	
48	mutant	IDH2 R172M	147,017	212	80931	16499557	4,91E-03	7,067	0,10	3,5	O, IDH-mutant	II	
49	mutant	IDH1 R132H	147,029	39	18018	53399393	3,37E-04	3,781	0,03	3,0	DA, IDH-mutant	II	
50	mutant	IDH1 R132H	147,021	34	23379	70721892	3,31E-04	5,263	0,20	2,0	AO, IDH-mutant	III	
51	mutant	IDH1 R132H	147,035	31	22517	72943304	3,09E-04	5,129	0,01	3,0	O, IDH-mutant	II	
52	mutant	IDH1 R132H	147,024	29	18186	39943127	4,55E-04	1,502	0,08	5,0	AO, IDH-mutant	III	
53	mutant	IDH1 R132H	147,035	21	12071	45173193	2,67E-04	0,839	0,07	2,0	O, IDH-mutant	II	
54	mutant	IDH1 R132H	147,043	41	52076	10454407	4,98E-03	2,974	0,15	3,0	O, IDH-mutant	II	
IDH mutant	mutant	IDH1 R132H	n.a	n.a	n.a	n.a	n.a	n.a	n.a	4,0	DA, IDH-mutant	II	used as IDH mutant test tissue
