# Supplementary figures and images for: Rapid detection of 2-hydroxyglutarate in frozen sections of IDH mutant tumors by MALDI-TOF mass spectrometry
Source: Acta Neuropathol Commun. 2018 Mar 2;6:21. doi: 10.1186/s40478-018-0523-3 (PMC5834865; doi:10.1186/s40478-018-0523-3)

**Figure S1**

**a**

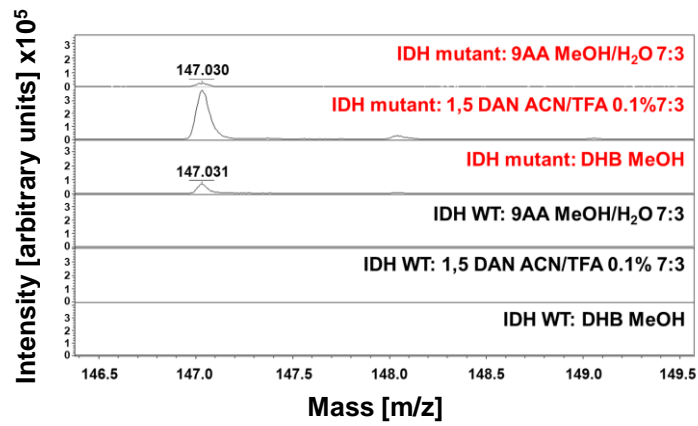

**b**

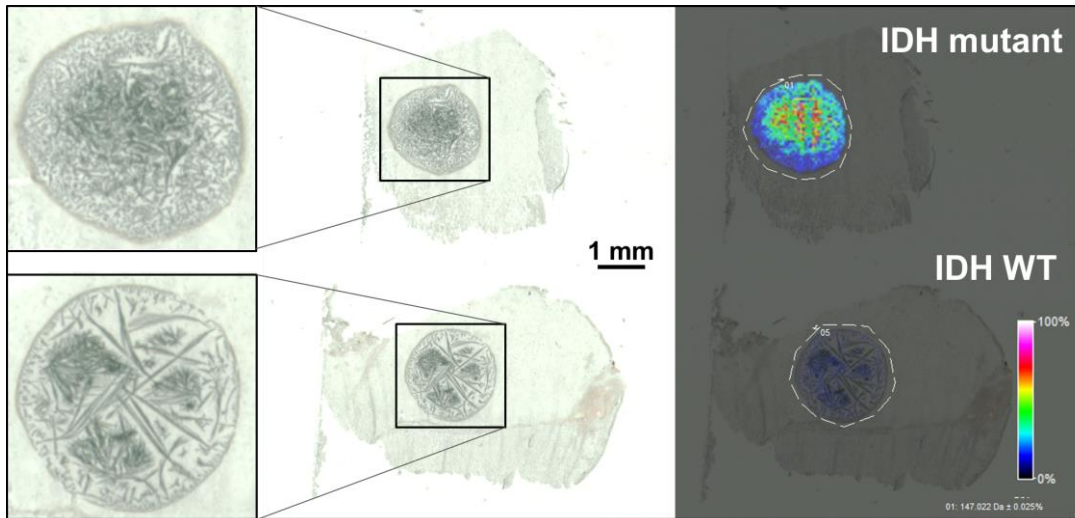

Supplement: Supplementary file 2 — Figure S1. Establishing of MALDI-TOF method. (a) MALDI-TOF spectra showing m/z values from 146.5 to 149.5 of solvent alone (black) and 10 mM D-2HG (red) with the matrices 9AA, 1,5-DAN and DHB. (b) Light microscopic image and corresponding MALDI-TOF scanned image of a 1 μl 1,5-DAN spot on IDH wildtype (lower) and IDH mutant (upper) tissue. The size bar represents 1 mm, the intensity bar shows the color coding for MALDI-TOF intensities within the scanned region. (PDF 196 kb) [file 40478_2018_523_MOESM2_ESM.pdf]
